# Supplementary material for: Cyclic GMP-AMP Synthase (cGAS) Deletion Promotes Less Prominent Inflammatory Macrophages and Sepsis Severity in Catheter-Induced Infection and LPS Injection Models
Source: Int J Mol Sci. 2025 May 24;26(11):5069. doi: 10.3390/ijms26115069 (PMC12154408; doi:10.3390/ijms26115069)
Supplement: Supplementary file 1 [file ijms-26-05069-s001.zip › ijms-3566469-supplementary.pdf]

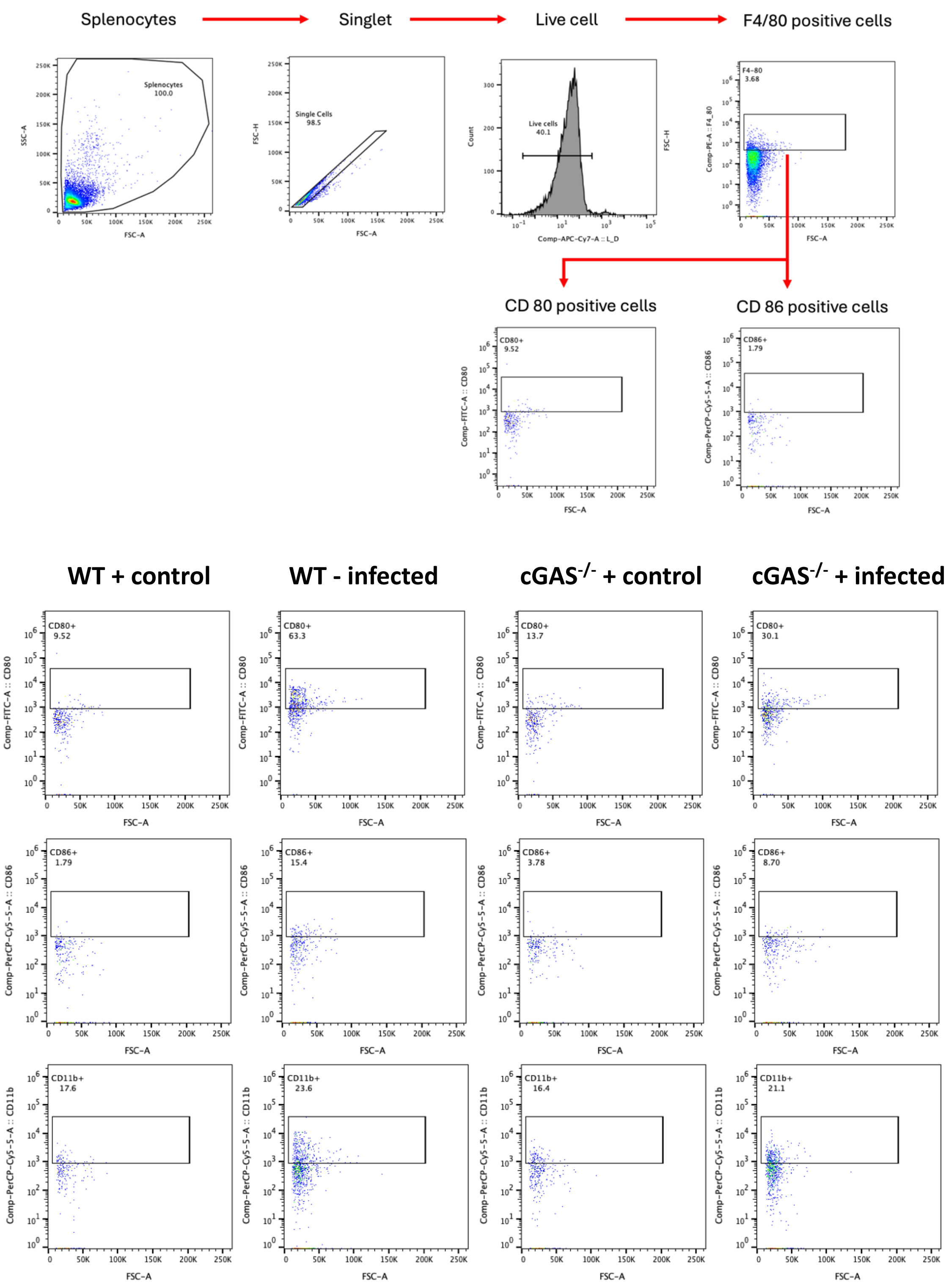

Figure S1. Flow cytometry gating strategy (upper panel) and representative positive cell populations (lower panel) of splenocytes from WT and cGAS$^{-/-}$ mice in a *P. aeruginosa*-infected, catheter-induced sepsis model.

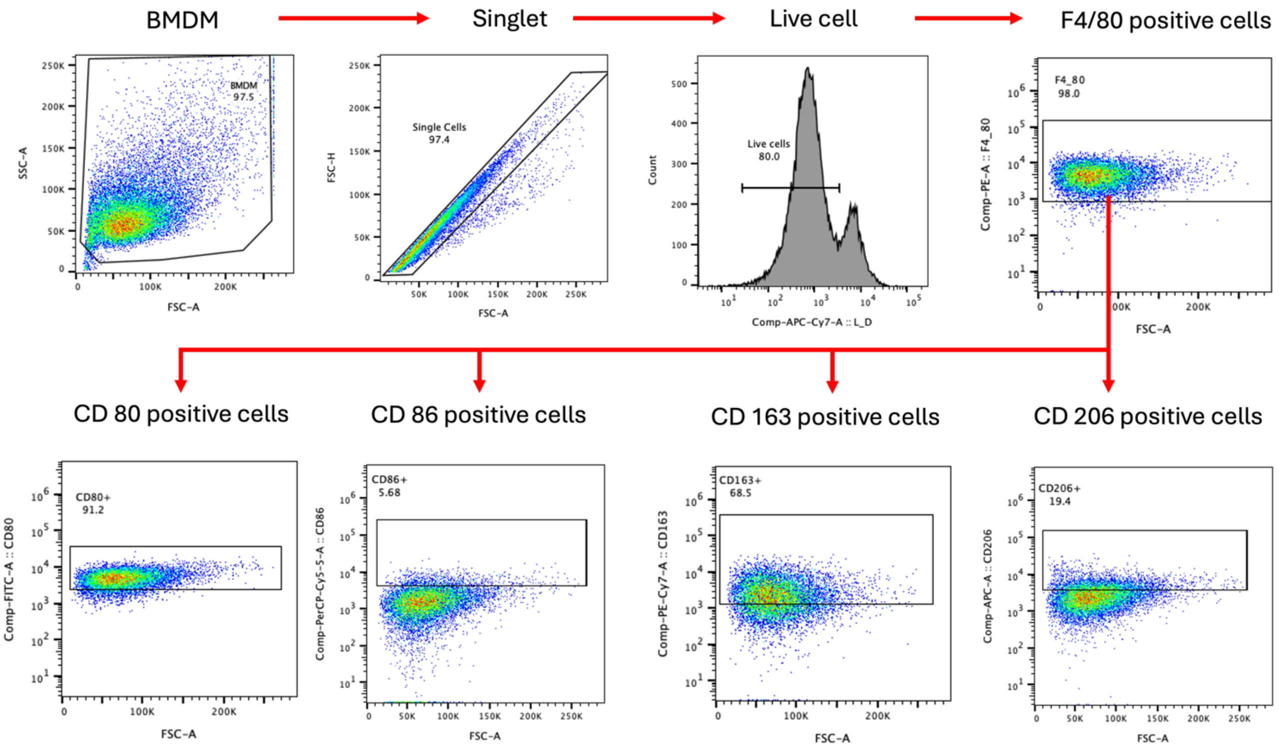

BMDM
Singlet
Live cell
F4/80 positive cells
BMDM
97.5
SSC-A
FSC-A
Single Cells
97.4
FSC-H
FSC-A
Live cells
80.0
Count
Comp-APC-Cy7-A :: L_D
F4_80
98.0
Comp-PE-A :: F4_80
FSC-A
CD 80 positive cells
CD 86 positive cells
CD 163 positive cells
CD 206 positive cells
CD80+
91.2
Comp-FITC-A :: CD80
FSC-A
CD86+
5.68
Comp-PerCP-Cy5-5-A :: CD86
FSC-A
CD163+
68.5
Comp-PE-Cy7-A :: CD163
FSC-A
CD206+
19.4
Comp-APC-A :: CD206
FSC-A

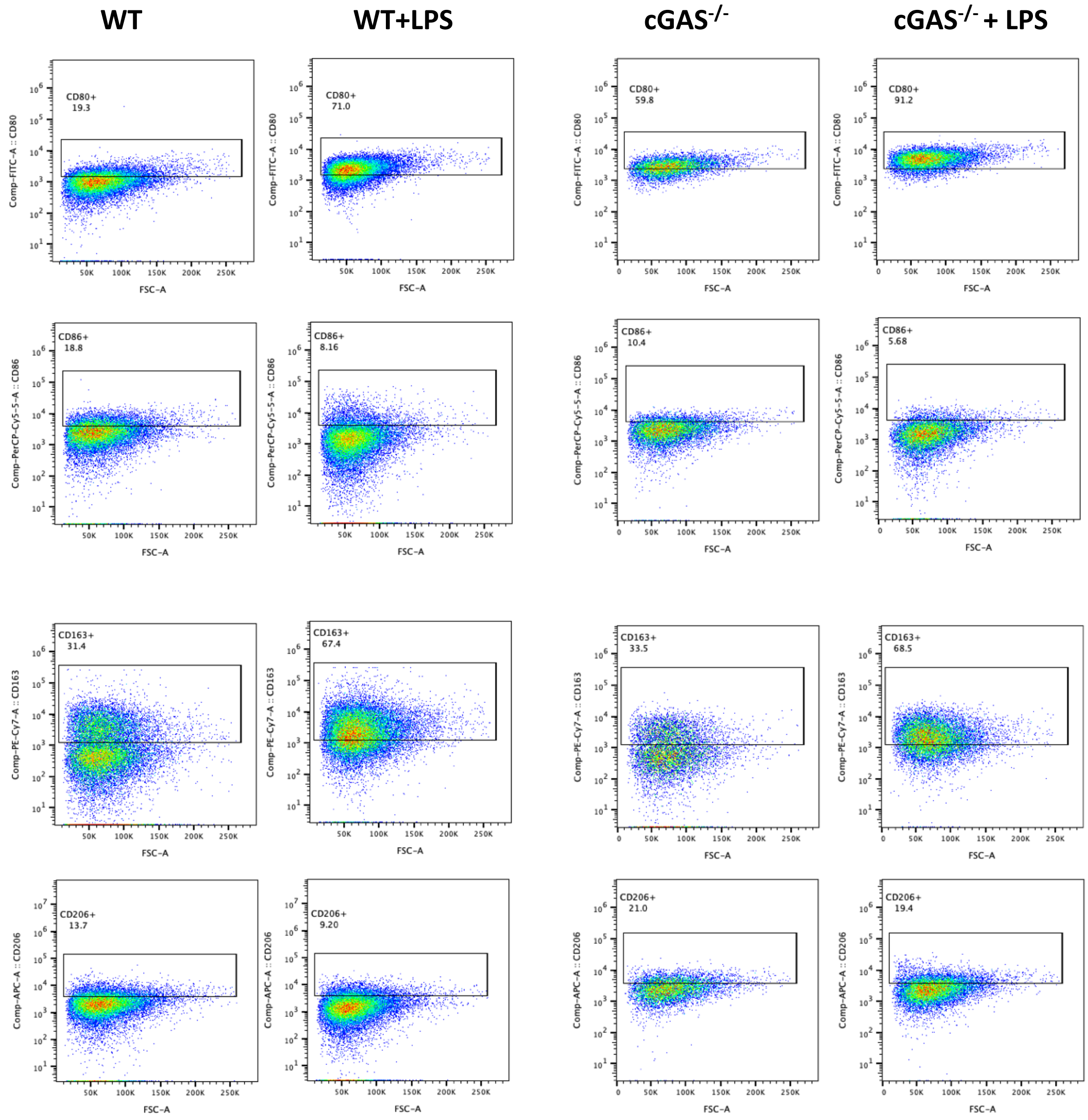

Figure S2. Flow cytometry gating strategy (upper panel) and representative expression of macrophage polarization markers (lower panel) in bone marrow-derived macrophages (BMDMs) from WT and cGAS$^{-/-}$ mice treated with LPS.

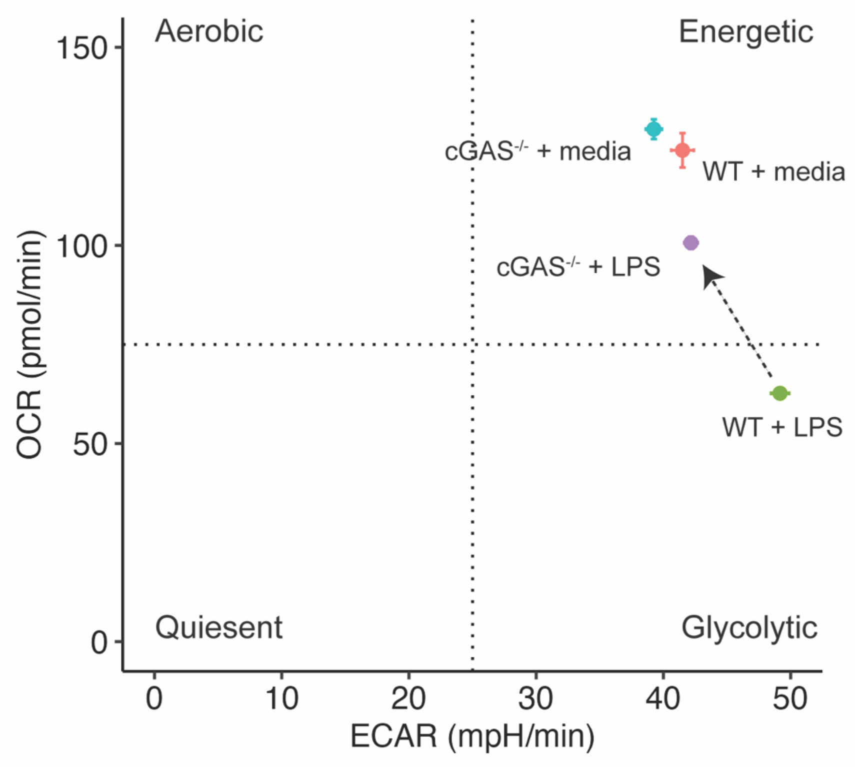

Figure S3. Bioenergetic profiles of wild-type (WT) and cGAS knockout ($cGAS^{-/-}$) bone marrow-derived macrophages (BMDMs) treated with media control or LPS. Data shown are the mean ± SEM (n = 6 biological replicates). Arrow indicates a shift of bioenergetic phenotype of BMDMs when cGAS is attenuated in LPS-treated condition.

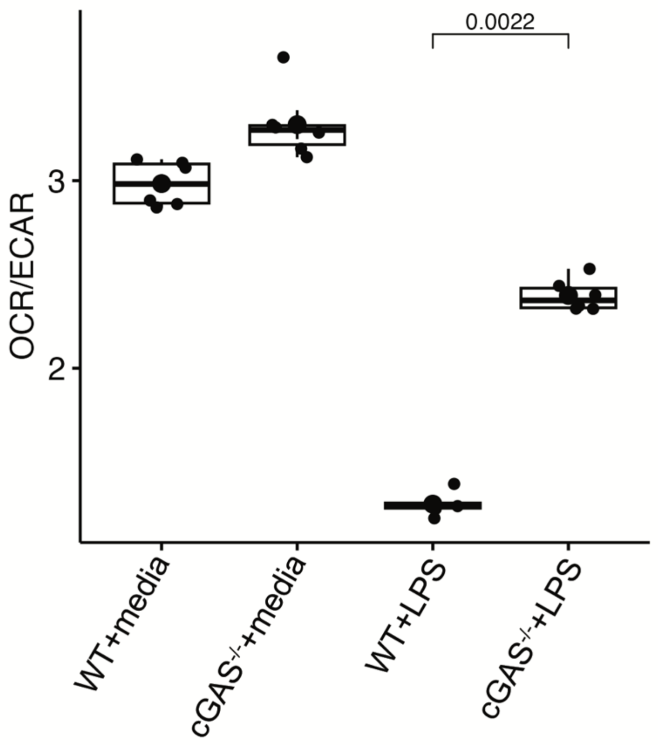

Figure S4. Ratio of OCR and ECAR of wild-type (WT) and cGAS knockout (cGAS$^{-/-}$) bone marrow-derived macrophages (BMDMs) after treatment with media control or LPS. The statistical significance of the difference between groups is determined by Wilcoxon test.

	Control catheter		Infected catheter	
	**WT**	**cGAS -/-**	**WT**	**cGAS -/-**
Serum TNF-α (pg/mL)	25 ± 2	28 ± 4	352 ± 73	161 ± 16
Serum IL-6 (pg/mL)	33 ± 4	27 ± 8	413 ± 16	246 ± 18
Serum IL-10 (pg/mL)	46 ± 4	48 ± 7	175 ± 18	109 ± 38

	Vehicle		LPS	
	**WT**	**cGAS -/-**	**WT**	**cGAS -/-**
Serum TNF-α (pg/mL)	21 ± 2	24 ± 3	1,698 ± 105	1,009 ± 38
Serum IL-6 (pg/mL)	32 ± 4	29 ± 8	124,229 ± 6,478	89,200 ± 4,507
Serum IL-10 (pg/mL)	49 ± 8	52 ± 10	75,386 ± 3,795	52,786 ± 7,340

Data presented with mean ± standard error

Table S.1 Serum cytokine levels in WT and $cGAS^{-/-}$ mice from a *P. aeruginosa*-infected, catheter-induced sepsis model (upper table) and an LPS-induced sepsis model (lower table)
